# Basal Cell Carcinoma Over Chest Wall (Sternum) Treated With Dufourmentel Flap: Report of a Case with Review of Literature

**DOI:** 10.4103/0974-2077.69026

**Published:** 2010

**Authors:** Vishal K Jain, Sachin S Verma, Archana S Verma, Kavita R Munjal, Bhavesh Swarnakar

**Affiliations:** *Department of Surgery, SAIMS Medical College, Indore, India*; 1 *Department of Plastic Surgery, Bombay Hospital, Indore, India*; 2 *Department of Medicine MGM Medical College, Indore, India*; 3 *Department of Pathology, SAIMS Medical College, Indore, India*; 4 *Department of Skin and Veneral Disease, Kaya Clinic, Indore, India*

**Keywords:** Basal cell carcinoma, dufourmentel flap, rhomboid transposition flap, sternum

## Abstract

Basal cell carcinoma (BCC) is the most common malignancy of the skin, accounting for approximately 70–80% of all cutaneous cancers. The commonest site of basal cell carcinoma is the face; 80% arise above a line from the corner of the mouth to the ear lobe. The lifetime ultraviolet radiation damage is the most important factor in its pathogenesis, and the vast majority is observed on sun-exposed skin. BCCs can develop in sun-protected areas, but its occurrence is rare. Here we are reporting a case of rare site of BCC with review of literature in a 65-year-old male who presented with a lesion over anterior chest wall. A clinical diagnosis of BCC was made and patient was subjected to excision biopsy. Biopsy revealed it to be a BCC and it was treated with a Dufourmentel flap.

## INTRODUCTION

Basal cell carcinoma (BCC) is the most common malignancy of the skin, accounting for approximately 70–80% of all cutaneous cancers.[1] The lifetime ultraviolet radiation damage is the most important factor in its pathogenesis, and the vast majority is observed on sun-exposed skin, with nearly 85% occurring in the head and neck.[2] Although BCCs can develop in sun-protected areas, genital involvement is very rare, accounting for fewer than 1% of all cases.[3] Here we are reporting anterior chest wall (sternal region) as a rare site of occurrence of BCC. The surgical management of the condition is also discussed.

## CASE REPORT

**Figure 1:**
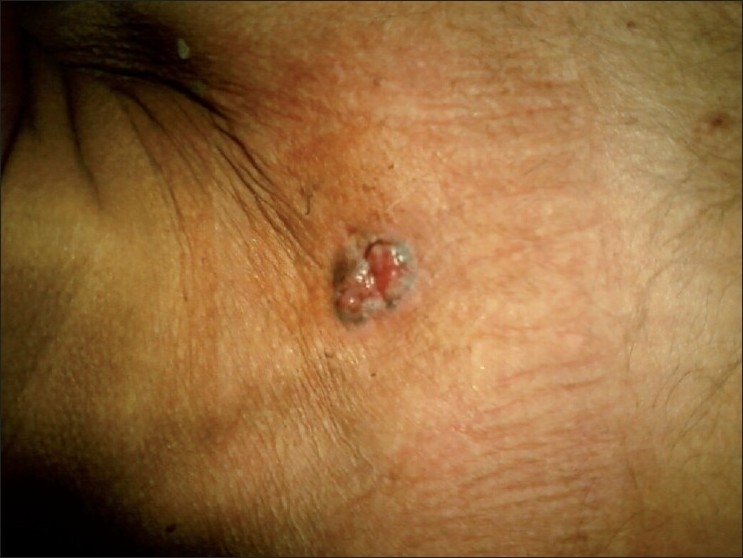
Basal cell carcinoma at sternum

A 65-year-old male patient presented to the department of dermatology with a lesion over his anterior chest wall (sternal region) of 2 years. It was painless, with no other associated symptoms like itching or bleeding. Lesion was gradually increasing in size. A provisional clinical diagnosis of BCC [Figure 1] was made and referred to surgery department for excision.

**Figure 2:**
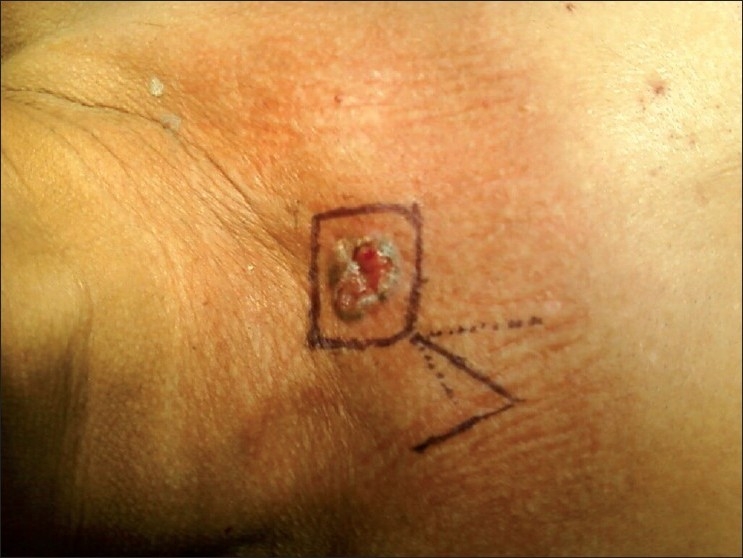
Marking for excision and flap planning

**Figure 3:**
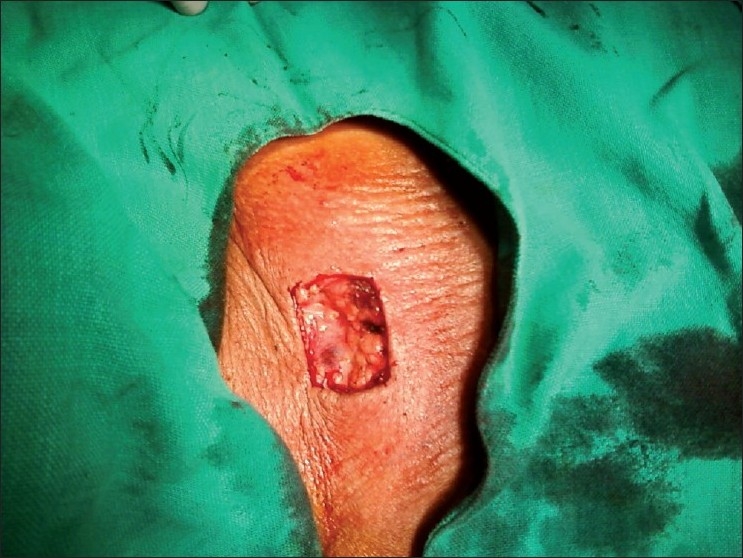
Defect created after excision

**Figure 4:**
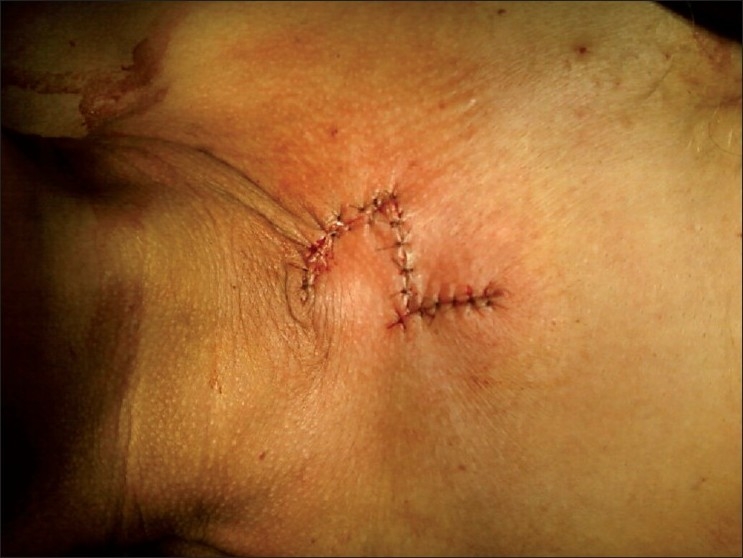
Defect covered with Dufourmentel flap

Patient was planned for surgery and was subjected to excision biopsy [Figure 2] under local anesthesia. Lesion was excised completely taking wide margins all around. A defect of 5 cm × 4 cm was created [Figure 3]. Being a large defect, it could not be closed with primary repair; therefore it was covered with local duformentel flap/rhomboid transposition flap [Figure 4]. Postoperative course was uneventful and stitches were removed on 10th postoperative day. Histopathology report revealed it to be a BCC of 2.5 cm × 2.5 cm × 1 cm with free margins. The patient is on regular follow up for 1 year and is currently asymptomatic.

## DISCUSSION

BCC is one of the commonest of all malignant tumors, but it accounts for less than 1% deaths due to malignant disease. It has a very low rate of metastasis - the incidence is ranging from 0.0028% to 0.54%.

There has been a progressive increase in the incidence of skin cancers, particularly that of cutaneous melanomas over the last few decades.[4]

Both BCC and squamous cell carcinoma (SCC) are common in whites but rare in blacks and Indians. Contrast to one-third malignancies of nonmelanoma skin cancer in whites, in Indians only 1–2% of cancer occurs on skin.[5] Also, these cancers mainly affect sun-exposed areas like neck and face (88–90%).[6]

BCC is the commonest form of skin cancer worldwide, but various studies from India have consistently reported SCC as the most prevalent skin malignancy.[7] Although complete data of incidence are not available, various cancer registries in India reported cumulative incidence of skin cancer varying from 0.5 to 2 per 100 000 population.[8]

### Sites

The commonest site of BCC is the face; 80% arise above a line from the corner of the mouth to the ear lobe. Occurrence of BCC on the scrotum is extremely rare, accounting for less than 0.05% to 0.19% of all BCC cases.[9–11] Very few cases of BCC on the pudendum have been reported in the literature.[9–13] Literatures cite posterior neck,[14] scrotum,[15] palm,[16] nipple,[17] areola,[18] shotgun scar,[19] buttock, perineum, axilla, genital region,[20] conjunctiva[21] and Submandibular gland[22] as unusual or rare site of BCC. We are reporting another rare site as anterior chest wall (sternal region). Only few cases have been reported in the literature of BCC on chest wall.[23,24]

### Treatment

Although, surgery is the mainstay of treatment for all the three common skin cancers, the extent of surgery, both local and regional, varies. Adequate surgery is most important to prevent recurrence. Adequate surgical margin is very important, particularly for melanoma, where margin depends upon the thickness (depth of infiltration) of cancer.[25]

Simple surgical excision is effective for all types of BCCs. The cure rate approaches 99% when the histological margins are clear. Recommended margin is 5 mm; recurrence is more when the margin of resection is less than 4 mm.[26,27] Moh’s micrographic surgery has been implied for recurrent lesions or those located in vital areas such as eyelid, digits, penis, nose, etc., but it requires a dedicated surgeon pathologist and onsite facility for pathology examination, which is not present is most of the centers.

Surgical margin and possibility of requirement of reconstruction are directly related to each other and there is always a critical trade-off between them. Any compromise of the adequacy of surgical margin increases the chances of recurrence. A reconstructive procedure is always preferred to a potentially suboptimal surgical excision.[28] Here in our case, we used dufourmental flap to close the defect.

### Dufourmentel/rhomboid flap

A rhombus is classically defined as an oblique-angled equilateral parallelogram, whereas a rhomboid differs in that it has uneven adjacent sides. The term rhomboid is frequently used in facial reconstruction literature to mean either rhombus like or to describe one of the popular transposition flaps used to repair rhombus-shaped defects.

In 1946, Limberg first described a technique for closing a 60° rhombus-shaped defect with a transposition flap. Dufourmentel modified this technique in 1962 to close defects with any acute angle. Webster published a third significant modification in 1978.[1] The Webster, or 30° flap, uses a 30° angulation of the distal flap end along with an M-plasty closure at the defect base.

Transposition flaps are useful when the size or shape of a lesion does not permit direct closure using a standard fusiform incision. For example, attempting to close a wide defect primarily requires an ellipse with either long limbs or blunt angles. Lengthy limbs create long scars and remove healthy tissue unnecessarily, whereas blunt ends often create an unpleasant standing-cone or dog-ear appearance.

Considerations when designing any local flap are lesion diameter, amount of normal skin that needs to be discarded, scar orientation with respect to relaxed skin tension lines, arc of skin rotation, and the vector of maximal tension after closure. Rhomboid flaps have been used in reconstruction of the cheek, temple, lips, ears, nose, chin, eyelids, and neck. The aesthetic and mechanical properties of these flaps, however, make them especially useful for reconstruction of small defects in the lower cheek, mid-cheek, and upper lip.

Rhomboid flaps are full-thickness local flaps with a random blood supply. Rather than depending on an axial blood vessel for nourishment, rhomboid flaps rely upon the dermal-subdermal plexus of blood vessels.

The surgeon must not violate the dermis when undermining this or any other random flap because the chances for partial or complete flap necrosis increases.

The role of adjuvant therapy is limited in skin cancers. Although radiotherapy can be used as primary mode of treatment for BCC and SCC located at certain sites such as the nose, lip, eyelid, and canthus, where surgery is either technically difficult or likely to yield poor cosmesis. Radiotherapy has a very limited role in the management of melanoma.[29] Postoperative radiotherapy is indicated in patients with advanced lesions, positive margins, lymph node metastasis, in-transit metastases in melanoma, and for palliation.[30]

## CONCLUSION

BCC is a common surface malignancy and thus more amenable not only to early detection, but also to a potential cure. Face remains the most common site of BCC. As in our Case, a high index of suspicion is required to diagnose BCC at rare sites like anterior chest wall. With the help of appropriately designed flaps like Dufourmentel flap, good primary closure of defect with excellent cosmetic results can be achieved.
